# Body fatness and breast cancer risk in women of African ancestry

**DOI:** 10.1186/1471-2407-13-475

**Published:** 2013-10-14

**Authors:** Elisa V Bandera, Urmila Chandran, Gary Zirpoli, Zhihong Gong, Susan E McCann, Chi-Chen Hong, Gregory Ciupak, Karen Pawlish, Christine B Ambrosone

**Affiliations:** 1 Rutgers Cancer Institute of New Jersey, Robert Wood Johnson Medical School, 195 Little Albany Street, New Brunswick, NJ, 08903, USA; 2 Rutgers School of Public Health, Piscataway, NJ, USA; 3 Department of Cancer Prevention & Control, Roswell Park Cancer Institute, Buffalo, NY, USA; 4 New Jersey State Cancer Registry, New Jersey Department of Health, Trenton, NJ, USA

**Keywords:** Breast cancer, Obesity, Body mass index, Body fat distribution, Waist circumference, Body composition, Percent body fat, African American

## Abstract

**Background:**

Obesity has been shown to be inversely associated with breast cancer risk in premenopausal women, while increasing risk in postmenopausal women. However, the current evidence is largely based on studies in Caucasian populations. Associations in women of African ancestry (AA), who have a higher prevalence of obesity, have been evaluated in few studies and results suggest different effects.

**Methods:**

We evaluated the impact of body size, body fat distribution, and body composition on breast cancer risk among AA women (978 cases and 958 controls) participating in the Women’s Circle of Health Study, a multi-site case–control study in New York City (NYC) and New Jersey (NJ). Cases were newly diagnosed with histologically confirmed ductal carcinoma *in situ* or invasive breast cancer, age 20–75 yrs. In NYC, cases were recruited through hospitals with the largest referral patterns for AA women and controls through random digit dialing (RDD). In NJ, cases were identified in seven counties in NJ thorough the NJ State Cancer Registry, and controls through RDD and community-based recruitment. During in-person interviews, questionnaires were administered and detailed anthropometric measurements were obtained. Body composition was assessed by bioelectrical impedance analysis.

**Results:**

BMI did not have a major impact on pre- or post-menopausal breast cancer, but was significantly associated with reduced risk of ER-/PR- tumors among postmenopausal women (OR: 0.37; 95% CI: 0.15-0.96 for BMI > 30 vs. BMI < 25). Furthermore, increased premenopausal breast cancer risk was found for higher waist and hip circumferences after adjusting for BMI, with ORs of 2.25 (95% CI: 1.07-4.74) and 2.91 (95% CI: 1.39-6.10), respectively, comparing the highest vs. lowest quartile. While ORs for higher fat mass and percent body fat among postmenopausal women were above one, confidence intervals included the null value.

**Conclusions:**

Our study suggests that in AA women BMI is generally unrelated to breast cancer. However, higher waist and hip circumferences were associated with increased pre-menopausal breast cancer risk, while general obesity was associated with decreased risk of ER-/PR- tumors. Larger studies are needed to confirm findings and to evaluate the impact of obesity on breast cancer subtypes.

## Background

In the United States, breast cancer is the most common cancer in women excluding skin cancer and the second leading cause of cancer mortality [1]. Rates vary considerably by race/ethnicity [1]. While women of African ancestry (AA) have lower incidence compared to those of European descent (EA), incidence is higher for those younger than 40 years [1]. AA women with breast cancer also experience the highest mortality rates for any racial/ethnic groups [1]. Several factors, many related to socio-economic status, have been proposed to explain these differences in AA women, including poorer access to screening, pre-existing conditions, suboptimal treatment for breast cancer, lifestyle factors, and obesity [2]. AA women tend to have aggressive tumor characteristics and more advanced tumors at diagnosis, which have also been linked to obesity [3,4].

Obesity is a major public health concern in AA women, with the prevalence of obesity (BMI ≥ 30) being 58.6% for “non-Hispanic black women” and 33.4% for “non-Hispanic white women”, according to 2009–2010 National Health and Nutrition Examination Survey (NHANES) data [5]. AA women also tend to have higher waist circumference than EA women [4]. Excess body fatness is a well-recognized risk factor for breast cancer, with studies showing increased risk for postmenopausal women and an inverse association in pre-menopausal women [6]. However, there is growing evidence that this relationship is complex, with the association varying by race, age, HRT use, and possibly by hormone receptor status [7]. Furthermore, the current evidence is largely based on studies conducted in white women, with only a few studies evaluating the role of measures of body fatness, such as body mass, central adiposity, or percent body fat, among AA women [7,8]. The evidence from the few studies that evaluated the impact of BMI on breast cancer risk in AA women is generally inconsistent but suggests different effects (reviewed in [3,7,8]). Although some studies have indicated increased breast cancer risk associated with higher waist circumference and waist-to-hip ratio in pre-menopausal women [9], the overall evidence independent of BMI remains uncertain. The impact of body composition on breast cancer risk in AA women is unknown. Therefore, we evaluated the impact of body mass index, body fat distribution and body composition on breast cancer risk among pre- and postmenopausal AA women participating in the Women’s Circle of Health Study.

## Methods

### Study population

The Women’s Circle of Health Study (WCHS) is a multi-site case–control study in New York City (NYC) and New Jersey (NJ) specifically designed to evaluate genetic and lifestyle risk factors for early/aggressive breast cancer and to compare the distribution of these factors in AA and EA women. The study design has been described in detail elsewhere [10]. In brief, cases were self-identified AA and EA women, 20–75 years of age, and able to understand and read English with no previous history of cancer other than non-melanoma skin cancer, and diagnosed within 9 months with primary, histologically confirmed invasive breast cancer or ductal carcinoma *in situ* (DCIS). Controls had the same eligibility criteria and no history of cancer and were identified, recruited, and interviewed during the same time period as the cases.

In NYC, cases were identified through collaborations with hospitals in Manhattan, Bronx, Brooklyn, and Queens with the largest referral patterns for AA women. Controls in NYC were ascertained by RDD, using the telephone exchanges (area code plus three-digit prefixes) of breast cancer cases who received medical care at the participating hospitals in previous years for sampling, frequency matched by 5-year age groups and race. In New Jersey, cases were identified through the NJ State Cancer Registry (NJSCR), using rapid case ascertainment in seven counties (Bergen, Essex, Hudson, Mercer, Middlesex, Passaic, and Union), with extension Monmouth and Burlington counties in 2012. Controls were identified by RDD in NYC and NJ, frequency matched by age group and county of residence to the cases. In NJ, controls were also recruited through community sources, as described in detail elsewhere [11].

### Data collection

After confirming eligibility, an in-person interview was scheduled at the participants’ homes or a mutually agreed upon location. All interviewers underwent rigorous standardized training and testing before they were allowed to start conducting interviews and anthropometric measurements. Informed consent was obtained in person at the visit before data collection began. During the in-person interview several questionnaires were completed, body measurements taken, and biospecimens collected.

The WCHS survey instrument covered established and suspected risk factors for breast cancer, including family history, reproductive and menstrual history, hormone use, alcohol intake and smoking, occupational history, and physical activity. Women were also asked to report their weight and height one year before diagnosis (for cases) or reference date (for controls), and at several times during their life.

Anthropometric measurements were taken at the end of the visit using a standardized protocol based on the Women’s Interview Study of Health [12] and measuring instruments. Participants were asked to wear light clothing and to remove their shoes and any heavy jewelry. Standing height was measured once to the nearest 0.1 cm. Waist and hip circumferences were measured twice to the nearest 0.1 cm by placing the measuring tape around the waist covering the umbilicus (for waist) or at the maximum extension of the buttocks (for hip) in a horizontal plane. If the difference between the first and second measurement was greater than 2 cm, a third measurement was taken. The two (or three) measurements were averaged for analyses. While there is no uniformly accepted protocol for measuring waist and hip circumference, repeated measurements have been shown to reduce measurement error and a clinically relevant change in waist circumference has been estimated to range between 3.0 and 6.8 cm [13]. Therefore, a tolerance limit of 2 cm, below this clinically relevant range, was used. Body composition (lean and fat mass, percent body fat) was measured by bioelectrical impedance analysis using a Tanita® TBF-300A scale. Weight was measured once using the Tanita scale.

A total of 979 AA cases and 958 AA controls completed the interview. Height and/or weight measurements were not available for 25 cases and 13 controls because the participants refused (n = 24), were more than 3 months pregnant (n = 2), or for other reasons (e.g., physical impairments) (n = 12). Because we found high correlation between BMI based on measurements and based on self-reported weight and height (r = 0.92), missing values for height and/or weight were substituted with self-reported values when available to compute the final BMI variable used to compute risk estimates. One case was excluded because she did not have measured or self-reported weight and height, leaving 978 cases and 958 controls for analyses.

The study was approved by the Institutional Review Boards at the Cancer Institute of New Jersey (now Rutgers Cancer Institute of New Jersey), Mount Sinai School of Medicine (now the Icahn School of Medicine at Mount Sinai), and Roswell Park Cancer Institute and all participants provided written informed consent before participating in the study.

### Statistical analyses

All analyses were conducted separately in pre- and postmenopausal women. BMI was computed as weight in kilograms (kg) divided by the square of height in meters (m). Self-reported BMI one year prior to reference date (diagnosis date for cases and comparable date for controls) was calculated using measured height at interview and self-reported weight, except for 26 women for whom measured height was missing and instead, self-reported height was used. Fat mass index and fat free mass index were calculated as fat mass or fat free mass in kg, respectively, divided by the square of height in meters. BMI was categorized according to the World Health Organization (WHO) International Classification. For all other variables we used quartiles, based on the distribution of all controls combined. We used the same cutpoints in the two subgroups by menopausal status to be able to compare risk estimates across categories.

Distributions of selected risk factors between cases and controls stratified by menopausal status were compared using chi-square tests and means compared using the *t*-test. Multivariable unconditional logistic regression was used to estimate odds ratios (OR) and 95% confidence intervals (CI), controlling for relevant confounders. Tests for trend were derived by assigning the median value to each category. Covariates considered included age, ethnicity (Hispanic vs. not Hispanic), country of origin (United States, Caribbean countries, other), education, family history of breast cancer, history of benign breast disease, age at menarche, age at menopause, parity, breastfeeding (never/ever), age at first birth, hormone replacement therapy use (never/ever), oral contraceptive use (never/ever), years since menopause (for analyses of body composition), and BMI in young adulthood. We also controlled for waist circumference when evaluating relationships with BMI, and BMI when evaluating relationships with body fat distribution to assess the potentially independent effects of general and central obesity, respectively.

Possible effect modification by hormone receptor status was evaluated by conducting stratified analyses according to the major subtypes (ER+/PR + and ER-/PR-) in addition to conducting case-case analyses to estimate risk of ER-/PR- tumors compared to ER+/PR+ tumors. Analyses were repeated excluding HRT users and non-invasive tumors, as well as community controls. A finding with a p value of less than 0.05 was considered statistically significant. SAS version 9.2 (SAS Institute, Cary NC) was used for analyses.

## Results

Selected characteristics for study participants by menopausal status are shown in Table 1. For both pre- and post-menopausal women, cases tended to be slightly older and have fewer years of education than controls, while family history of breast cancer, history of benign breast disease, and use of HRT were more common in cases than controls in both pre- and post-menopausal women.

**Table 1:** Selected characteristics among women of African ancestry in the Women’s Circle of Health Study

	**Pre-menopausal**			**Post-menopausal**		
	**Cases**	**Controls**	**p value**	**Cases**	**Controls**	**p value**
	**(n = 469)**	**(n = 482)**		**(n = 509)**	**(n = 476)**	
	**n (%)**	**n (%)**		**n (%)**	**n (%)**	
**Age (yrs)**						
Mean ± SD	43.93 ± 6.8	42.65 ± 7.1	0.005	59.68 ± 6.7	57.70 ± 6.1	<0.0001
**Education**						
<High school	57 (12.2)	67 (13.9)	0.07	90 (17.7)	57 (12)	0.003
High school graduate	133 (28.4)	112 (23.2)		165 (32.4)	130 (27.3)	
Some college	136 (29)	120 (24.9)		122 (24)	159 (33.4)	
College graduate	91 (19.4)	121 (25.1)		81 (15.9)	74 (15.6)	
Post-graduate degree	52 (11.1)	62 (12.9)		51 (10)	56 (11.8)	
**Marital Status**						
Married	184 (39.2)	176 (36.5)	0.05	158 (31.1)	155 (32.7)	0.19
Living as married	8 (1.7)	9 (1.9)		6 (1.2)	11 (2.3)	
Widowed	10 (2.1)	14 (2.9)		88 (17.3)	57 (12)	
Separated	35 (7.5)	22 (4.6)		38 (7.5)	38 (8)	
Divorced	71 (15.1)	55 (11.4)		102 (20.1)	93 (19.6)	
Single	161 (34.3)	206 (42.7)		116 (22.8)	120 (25.3)	
**Country of origin**						
United States	297 (63.3)	378 (78.4)	<0.0001	404 (79.4)	391 (82.1)	0.54
Caribbean	131 (27.9)	75 (15.6)		74 (14.5)	61 (12.8)	
Other	41 (8.7)	29 (6)		31 (6.1)	24 (5)	
**Ethnicity**						
Hispanic	29 (6.2)	14 (2.9)	0.02	16 (3.1)	12 (2.5)	0.56
Non-Hispanic	440 (93.8)	468 (97.1)		493 (96.9)	464 (97.5)	
**Age at menarche (yrs)**						
<12	139 (29.6)	144 (29.9)	0.52	152 (29.9)	121 (25.5)	0.23
12-13	220 (46.9)	211 (43.8)		221 (43.4)	229 (48.2)	
>13	110 (23.5)	127 (26.4)		136 (26.7)	125 (26.3)	
**Age at menopause (yrs)**						
≤45				70 (14.1)	78 (16.5)	<0.001
46-49				64 (12.9)	107 (22.7)	
50-54				311 (62.5)	242 (51.3)	
≥55				53 (10.6)	45 (9.5)	
**Parity (livebirths)**						
0	87 (18.6)	82 (17)	0.17	67 (13.2)	75 (15.8)	0.17
1-2	257 (54.8)	241 (50)		242 (47.5)	235 (49.4)	
3-4	104 (22.2)	127 (26.4)		137 (26.9)	126 (26.5)	
≥5	21 (4.5)	32 (6.6)		63 (12.4)	40 (8.4)	
**Age at first birth (yrs)**						
Nulliparous	87 (18.6)	82 (17)	0.32	67 (13.2)	75 (15.8)	0.41
≤19	127 (27.1)	148 (30.7)		190 (37.3)	168 (35.4)	
20-24	108 (23.1)	117 (24.3)		131 (25.7)	120 (25.3)	
25-30	89 (19)	70 (14.5)		82 (16.1)	64 (13.5)	
≥31	57 (12.2)	65 (13.5)		39 (7.7)	47 (9.9)	
**Breastfeeding**						
Nulliparous	87 (18.6)	82 (17)	0.32	67 (13.2)	75 (15.8)	0.34
Never (among parous)	158 (33.7)	185 (38.4)		267 (52.5)	230 (48.3)	
Ever	224 (47.8)	215 (44.6)		175 (34.4)	171 (35.9)	
**Family history**						
No	370 (78.9)	416 (86.3)	0.003	365 (71.7)	389 (81.7)	<0.001
Yes	99 (21.1)	66 (13.7)		144 (28.3)	87 (18.3)	
**History of benign breast disease**						
Never	333 (71.3)	393 (81.7)	<0.001	330 (64.8)	345 (72.5)	0.01
Ever	134 (28.7)	88 (18.3)		179 (35.2)	131 (27.5)	
**HRT use**						
Never	449 (96.2)	467 (96.9)	0.53	375 (74.4)	378 (79.8)	0.05
Ever	18 (3.9)	15 (3.1)		129 (25.6)	96 (20.3)	
**Oral contraceptive use**						
Never	175 (37.3)	189 (39.2)	0.55	212 (41.7)	220 (46.4)	0.14
Ever	294 (62.7)	293 (60.8)		296 (58.3)	254 (53.6)	
**Smoking status**						
Never smoker	297 (71.4)	287 (60.7)	<0.001	219 (53.4)	226 (52.3)	0.13
Former smoker	62 (14.9)	76 (16.1)		127 (31)	117 (27.1)	
Current smoker	57 (13.7)	110 (23.3)		64 (15.6)	89 (20.6)	

*SD* standard deviation.

As shown in Table 2, there was little indication that BMI played a major role in breast cancer risk in AA women. ORs for both pre- and post-menopausal women with BMI > 40 were below one, but the confidence intervals included the null. We also evaluated BMI based on self-reported weight one year before reference date and measured height at interview, with no clear evidence of a dose–response association either. Results were similar after adjusting for waist circumference (Table 2).

**Table 2:** Association of body size and breast cancer risk in women of African ancestry, Women’s Circle of Health Study

	**Pre-menopausal**					**Post-menopausal**				
	**Cases/Controls**	**OR1**	**95% CI**	**OR2**	**95% CI**	**Cases/Controls**	**OR1**	**95% CI**	**OR2**	**95% CI**
Current BMI*										
Underweight/Normal (<25)	102/103	Ref		Ref		74/68	Ref		Ref	
Overweight (25–29.99)	150/142	1.02	0.70–1.49	1.05	0.70–1.57	131/132	0.93	0.60–1.44	0.93	0.59–1.47
Obese (≥30)	217/237	0.89	0.61–1.28	0.92	0.54–1.56	304/276	0.98	0.66–1.45	1.00	0.58–1.72
p for trend			*0.40*		*0.65*			*0.94*		*0.86*
Current BMI*										
Underweight/Normal (<25)	102/103	Ref		Ref		74/68	Ref		Ref	
Pre–obese (25–29.99)	150/142	1.01	0.69–1.48	0.98	0.64–1.49	131/132	0.93	0.60–1.44	0.88	0.55–1.41
Obese Class I (30–34.99)	111/94	1.06	0.70–1.61	0.93	0.54–1.62	146/121	1.08	0.70–1.68	0.98	0.56–1.72
Obese Class II (35–39.99)	50/68	0.71	0.43–1.17	0.62	0.31–1.26	94/89	0.90	0.56–1.44	0.80	0.41–1.58
Obese Class III (≥40)	56/75	0.79	0.49–1.29	0.64	0.26–1.56	64/66	0.89	0.53–1.48	0.74	0.31–1.78
p for trend			*0.17*		*0.20*			*0.65*		*0.47*
BMI one year before reference date**										
Underweight/Normal (<25)	115/113	Ref		Ref		66/77	Ref		Ref	
Overweight (25–29.99)	138/143	0.86	0.59–1.25	0.90	0.61–1.33	147/140	1.27	0.82–1.96	1.32	0.84–2.07
Obese (≥30)	214/221	0.91	0.63–1.30	1.00	0.62–1.62	293/254	1.27	0.85–1.89	1.46	0.87–2.44
p for trend			*0.74*		*0.88*			*0.41*		*0.22*
BMI one year before reference date**										
Underweight/Normal (<25)	115/113	Ref		Ref		66/77	Ref		Ref	
Pre-obese (25–29.99)	138/143	0.86	0.59–1.25	0.87	0.58–1.29	147/140	1.27	0.82–1.96	1.32	0.83–2.08
Obese Class I (30–34.99)	121/92	1.17	0.77–1.76	1.16	0.71–1.90	143/129	1.23	0.79–1.90	1.34	0.80–2.27
Obese Class II (35–39.99)	43/73	0.55	0.33–0.90	0.54	0.28–1.02	87/55	1.70	1.02–2.82	1.97	1.02–3.82
Obese Class III (≥40)	50/56	0.93	0.56–1.54	0.93	0.43–2.00	63/70	0.98	0.58–1.64	1.19	0.55–2.55
p for trend			*0.39*		*0.61*			*0.86*		*0.64*

*BMI measured at interview. **Based on self-reported weight one year before reference date and measured height at interview.

OR1: Adjusted for age, ethnicity, country of origin (United States, Caribbean, other), education, family history of breast cancer, history of benign breast disease, age at menarche, age at menopause (for postmenopausal women), parity (continuous), breastfeeding (yes/no), age at first birth, HRT use (ever/never), OC use (ever/never). OR2: Further adjusted for waist circumference.

When we evaluated measures of body fat distribution in pre- and post-menopausal women (Table 3), there was a weak positive association for postmenopausal women with higher waist-to-hip ratio (WHR) but the confidence intervals included the null value (OR: 1.43; 95% CI: 0.95-2.18 comparing highest to lowest quartile). However, after adjusting for BMI, a strong association emerged with waist and hip circumferences for pre-menopausal women, with an OR for waist circumference of 2.25 (95% CI: 1.07-4.74) for the highest vs. lowest quartile and an estimated 10% increase in risk per 5 cm increment in waist circumference. For hip circumference, after adjusting for BMI, the OR for the highest vs. lowest quartile was 2.91 (95% CI: 1.39-6.10), with a 12% increase in risk per 5 cm increment in hip circumference. Among post-menopausal women, no clear associations were observed. No association was also found for pre- or postmenopausal women with any of the body composition measures considered: fat mass, fat free mass, percent body fat, fat mass index, and fat free mass index (Table 4).

**Table 3:** Body fat distribution measures and breast cancer risk

	**Pre-menopausal**					**Post-menopausal**				
	**Cases/Controls**	**OR1**	**95% CI**	**OR2**	**95% CI**	**Cases/Controls**	**OR1**	**95% CI**	**OR2**	**95% CI**
**Waist to hip ratio**										
≤0.82	123/144	Ref		Ref		65/93	Ref		Ref	
0.83–0.87	128/120	1.14	0.79–1.65	1.20	0.82–1.74	146/117	1.58	1.03–2.40	1.59	1.04–2.42
0.88–0.92	105/121	0.98	0.66–1.44	1.07	0.72–1.60	120/116	1.22	0.79–1.88	1.24	0.80–1.92
>0.92	104/93	1.19	0.80–1.79	1.38	0.89–2.12	169/144	1.43	0.95–2.18	1.48	0.97–2.26
p for trend			*0.55*		*0.22*			*0.33*		*0.27*
**Waist circumference (cm)**										
≤87.88	137/143	Ref		Ref		87/94	Ref		Ref	
87.89–97.75	124/119	1.02	0.70–1.46	1.26	0.85–1.88	119/118	1.07	0.71–1.61	1.13	0.73–1.76
97.76–110.25	107/116	0.93	0.64–1.37	1.47	0.88–2.44	154/121	1.35	0.90–2.02	1.51	0.92–2.48
>110.25	92/100	0.98	0.65–1.48	2.25	1.07–4.74	140/137	1.00	0.67–1.50	1.23	0.64–2.34
p for trend			*0.84*		*0.04*			*0.96*		*0.48*
Per 5 cm		0.99	0.95–1.03	1.10	1.00–1.22		1.00	0.96–1.04	1.03	0.94–1.13
**Hip circumference (cm)**										
≤103.18	127/138	Ref		Ref		107/99	Ref		Ref	
103.19–111.63	133/111	1.25	0.86–1.81	1.60	1.07–2.39	129/126	0.99	0.67–1.47	0.99	0.65–1.51
111.64–123.15	101/116	1.00	0.68–1.46	1.60	0.98–2.60	145/122	1.16	0.79–1.72	1.16	0.71–1.89
>123.15	100/113	1.07	0.72–1.59	2.91	1.39–6.10	119/123	0.88	0.59–1.31	0.87	0.45–1.71
p for trend			*0.99*		*0.01*			*0.54*		*0.69*
Per 5 cm		0.98	0.93–1.03	1.12	0.99–1.27		0.98	0.94–1.03	0.97	0.87–1.08

OR1: Adjusted for age, ethnicity, country of origin (United States, Caribbean, other), education, family history of breast cancer, history of benign breast disease, age at menarche, age at menopause (for postmenopausal women), parity (continuous), breastfeeding (yes/no), age at first birth, HRT use (ever/never), OC use (ever/never). OR2: Further adjusted for BMI.

**Table 4:** Body composition measures and breast cancer

	**Pre-menopausal**					**Post-menopausal**				
	**Cases/Controls**	**OR1**	**95% CI**	**OR2**	**95% CI**	**Cases/Controls**	**OR1**	**95% CI**	**OR2**	**95% CI**
**Fat mass (kg)**										
≤25.3	128/125	Ref		Ref		101/102	Ref		Ref	
25.4–34.1	131/116	1.08	0.74–1.56	1.24	0.82–1.87	126/107	1.16	0.78–1.74	1.34	0.86–2.07
34.2–44.3	96/100	0.92	0.62–1.37	1.19	0.70–2.03	124/123	0.94	0.63–1.40	1.21	0.72–2.04
>44.3	87/114	0.82	0.54–1.23	1.39	0.61–3.14	120/110	1.05	0.70–1.57	1.69	0.82–3.49
p for trend			*0.24*		*0.49*			*0.93*		*0.22*
**Fat free mass (kg)**										
≤44.1	112/108	Ref		Ref		130/118	Ref		Ref	
44.2–48	121/125	0.94	0.64–1.39	1.04	0.70–1.55	134/99	1.28	0.87–1.88	1.32	0.88–1.96
48.1–52.6	106/99	1.00	0.67–1.51	1.24	0.78–1.98	107/124	0.87	0.59–1.27	0.92	0.59–1.43
>52.6	103/123	0.84	0.57–1.26	1.32	0.70–2.47	101/101	0.96	0.65–1.43	1.05	0.61–1.83
p for trend			*0.44*		*0.35*			*0.47*		*0.88*
**Percent body fat**										
≤35.9	145/137	Ref		Ref		93/94	Ref		Ref	
36–41.6	118/118	0.92	0.64–1.33	1.02	0.69–1.52	123/107	1.14	0.75–1.71	1.27	0.83–1.96
41.7–46.5	96/104	0.88	0.60–1.30	1.07	0.67–1.72	119/124	0.85	0.57–1.29	1.05	0.65–1.70
>46.5	83/106	0.82	0.55–1.23	1.22	0.62–2.42	137/119	1.09	0.73–1.63	1.59	0.87–2.93
p for trend			*0.32*		*0.65*			*0.98*		*0.26*
**Fat mass index**										
≤9.37	128/129	Ref		–	–	86/96	Ref		-	-
9.38–12.78	138/119	1.09	0.75–1.58	-	-	124/104	1.39	0.92–2.11	-	-
12.79–16.77	93/98	0.95	0.63–1.42	-	-	140/127	1.13	0.75–1.70	-	-
>16.77	83/109	0.80	0.53–1.21	-	-	121/115	1.10	0.72–1.66	-	-
p for trend			*0.35*	-	-			*0.58*	-	-
**Fat free mass index**				-	-				-	-
≤16.64	126/118	Ref		-	-	123/106	Ref		-	-
16.65-18.04	116/121	0.88	0.60–1.28	-	-	108/104	0.94	0.63–1.40	-	-
18.05–19.80	99/99	0.85	0.57–1.28	-	-	129/124	0.89	0.60–1.30	-	-
>19.80	101/117	0.81	0.54–1.21	-	-	112/108	0.86	0.58–1.28	-	-
p for trend			*0.33*	-	-			*0.45*	-	-

Fat mass index: fat mass in kg/(height in m)^2^; Fat Free Mass index: fat free mass in kg/(height in m)^2^ OR1: Adjusted for age, ethnicity, country of origin, education, family history of breast cancer, history of benign breast disease, age at menarche, years since menopause (for postmenopausal women), parity (continuous), breastfeeding (yes/no), age at first birth, HRT use (never/ever), OC use (never/ever). OR2: Further adjusted for BMI.

Restricting analyses to invasive cases led to essentially the same results (data not shown). Because it has been suggested that the relationship between BMI and breast cancer risk may be limited to non-HRT users [14,15], we also repeated analyses excluding HRT users (Table 5). Among non-HRT users, while there was no association with BMI, other adiposity measures tended to increase risk but most estimates were not significant, with the exception of hip circumference among pre-menopausal women, which significantly increased breast cancer risk. Excluding community controls did not substantially change estimates.

**Table 5:** Association of body size and breast cancer risk among non-HRT users (excluding n = 267 HRT users)

	**Pre-menopausal**					**Post-menopausal**				
	**Cases/Controls**	**OR1**	**95% CI**	**OR2**	**95% CI**	**Cases/Controls**	**OR1**	**95% CI**	**OR2**	**95% CI**
Current BMI*										
Underweight/Normal (<25)	96/102	Ref		Ref		55/53	Ref		Ref	
Overweight (25–29.99)	147/136	1.09	0.74–1.61	1.12	0.74–1.69	86/107	0.76	0.46–1.26	0.77	0.46–1.31
Obese (≥30)	206/229	0.91	0.63–1.32	0.92	0.54–1.58	234/218	0.97	0.62–1.52	1.05	0.57–1.95
p for trend			*0.41*		*0.60*			*0.56*		*0.49*
**% Body Fat**										
≤35.9	139/133	Ref		Ref		69/73	Ref		Ref	
36–41.6	112/114	0.90	0.62–1.31	0.98	0.65–1.46	85/86	1.05	0.66–1.68	1.18	0.72–1.95
41.7–46.5	94/101	0.89	0.60–1.32	1.03	0.63–1.66	90/95	0.87	0.54–1.39	1.06	0.61–1.84
>46.5	79/103	0.81	0.53–1.22	1.08	0.54–2.17	102/96	1.03	0.65–1.64	1.49	0.74–2.99
p for trend			*0.31*		*0.88*			*0.94*		*0.38*
**Waist to hip ratio**										
≤0.82	118/144	Ref		Ref		52/70	Ref		Ref	
0.83–0.87	122/113	1.21	0.83–1.77	1.27	0.87–1.86	97/94	1.16	0.72–1.88	1.17	0.72–1.90
0.88–0.92	100/116	1.02	0.69–1.52	1.11	0.74–1.67	78/88	0.97	0.58–1.59	0.99	0.60–1.64
>0.92	100/90	1.22	0.80–1.84	1.38	0.89–2.14	140/121	1.29	0.81–2.06	1.34	0.83–2.16
p for trend			*0.51*		*0.22*			*0.35*		*0.28*
**Waist circumference (cm)**										
≤87.88	130/140	Ref		Ref		62/75	Ref		Ref	
87.89–97.75	120/114	1.04	0.71–1.50	1.25	0.83–1.88	82/91	1.04	0.64–1.67	1.18	0.70–1.97
97.76–110.25	102/112	0.94	0.64–1.39	1.40	0.83–2.35	114/98	1.39	0.87–2.20	1.74	0.99–3.06
>110.25	88/97	0.98	0.64–1.50	2.01	0.95–4.29	109/109	1.08	0.68–1.72	1.64	0.77–3.50
p for trend			*0.84*		*0.08*			*0.61*		*0.14*
**Hip circumference (cm)**										
≤103.18	120/134	Ref		Ref		76/81	Ref		Ref	
103.19–111.63	129/107	1.28	0.88–1.86	1.60	1.06–2.41	89/98	1.06	0.68–1.68	1.14	0.70–1.86
111.64–123.15	95/113	0.97	0.65–1.43	1.50	0.91–2.46	112/98	1.31	0.84–2.04	1.47	0.85–2.56
>123.15	97/109	1.10	0.74–1.65	2.78	1.32–5.88	90/96	0.97	0.61–1.54	1.22	0.56–2.62
p for trend			*0.92*		*0.02*			*0.92*		*0.65*

*BMI measured at interview.

OR1: Adjusted for age, ethnicity, country of origin (United States, Caribbean, other), education, family history of breast cancer, history of benign breast disease, age at menarche, age at menopause (for postmenopausal women), parity (continuous), breastfeeding (yes/no), age at first birth, OC use (ever/never). OR2: BMI further adjusted for waist circumference. Percent body fat, waist circumference and hip circumference, further adjusted for BMI. Percent body fat adjusted for years since menopause instead of age at menopause.

We also explored possible effect modification by the major breast cancer subtypes based on hormone receptor status: ER+/PR+ and ER-/PR- (Tables 6 and 7). The major findings were a strong association with hip circumference for ER+/PR+ tumors for pre-menopausal women and an inverse association with BMI for ER-/PR- tumors for post-menopausal women. No other clear patterns emerged from these analyses.

**Table 6:** Association of body size with premenopausal breast cancer by hormone receptor status

				**ER+/PR+ vs. controls**		**ER-/PR- vs. controls**		**ER-/PR- cases vs. ER+/PR+ cases**	
	***CO***	***CA***	**OR**	**95% CI**	***CA***	**OR**	**95% CI**	**OR**	**95% CI**
Current BMI*									
Underweight/Normal (<25)	103	42	Ref		20	Ref		Ref	
Overweight (25–29.99)	142	58	1.15	0.67–1.96	31	1.17	0.59–2.34	1.05	0.47–2.37
Obese (≥30)	237	95	1.34	0.68–2.68	51	1.26	0.53–3.00	0.88	0.30–2.54
p for trend				*0.40*			*0.65*		*0.74*
**% Body fat**									
≤35.9	137	62	Ref		20	Ref		Ref	
36–41.6	118	51	0.99	0.59–1.64	26	1.20	0.61–2.34	1.23	0.58–2.64
41.7–46.5	104	43	1.10	0.59–2.05	21	1.28	0.58–2.86	1.17	0.47–2.92
>46.5	106	30	0.91	0.37–2.24	24	1.78	0.59–5.37	1.68	0.47–6.08
p for trend				*0.96*			*0.36*		*0.52*
**Waist to hip ratio**									
≤0.82	144	56	Ref		20	Ref		Ref	
0.83–0.87	120	48	0.99	0.60–1.63	27	1.13	0.61–2.10	1.20	0.58–2.49
0.88–0.92	121	45	1.06	0.63–1.78	23	1.02	0.52–2.00	0.97	0.44–2.15
>0.92	93	43	1.33	0.76–2.32	24	1.51	0.74–3.05	1.30	0.57–3.00
p for trend				*0.33*			*0.32*		*0.63*
**Waist circumference (cm)**									
≤87.88	143	57	Ref		20	Ref		Ref	
87.89–97.75	119	52	1.28	0.76–2.17	24	1.07	0.55–2.10	0.80	0.37–1.75
97.76–110.25	116	47	1.57	0.80–3.08	25	1.52	0.67–3.47	0.78	0.28–2.16
>110.25	100	36	2.05	0.79–5.35	23	1.91	0.59–6.13	0.73	0.17–3.08
p for trend				*0.13*			*0.25*		*0.67*
**Hip circumference (cm)**									
≤103.18	138	55	Ref		20	Ref		Ref	
103.19–111.63	111	52	1.50	0.89–2.54	33	2.25	1.16–4.37	1.36	0.61–3.00
111.64–123.15	116	40	1.69	0.89–3.20	22	1.65	0.73–3.73	0.75	0.28–1.99
>123.15	113	46	3.59	1.43–9.03	24	2.60	0.79–8.54	0.47	0.12–1.95
p for trend				*0.01*			*0.22*		*0.15*

*BMI measured at interview.

*Abbreviations*: CO number of controls, CA number of cases, ER estrogen receptor, PR progesterone receptor.

Adjusted for age, ethnicity, country of origin (United States, Caribbean, other), education, family history of breast cancer, history of benign breast disease, age at menarche, parity (continuous), breastfeeding (yes/no), age at first birth, HRT use (ever/never), OC use (ever/never). BMI also adjusted for waist circumference. Percent body fat, waist circumference, and hip circumference, also adjusted for BMI.

**Table 7:** Association of body size with postmenopausal breast cancer by hormone receptor status

		**ER+/PR+ vs. controls**			**ER-/PR- vs. controls**			**ER-/PR- cases vs. ER+/PR+ cases**	
	***CO***	***CA***	**OR**	**95% CI**	***CA***	**OR**	**95% CI**	**OR**	**95% CI**
Current BMI*									
Underweight/Normal (<25)	68	26	Ref		20	Ref		Ref	
Overweight (25-29.99)	132	49	1.05	0.55–1.98	34	0.77	0.38–1.59	0.88	0.36–2.13
Obese (≥30)	276	131	1.04	0.50–2.18	47	0.37	0.15–0.96	0.42	0.14–1.25
p for trend				*0.95*			*0.03*		*0.07*
**% Body fat**									
≤35.9	94	34	Ref		23	Ref		Ref	
36–41.6	107	39	1.04	0.57–1.89	29	1.41	0.70–2.85	1.37	0.58–3.22
41.7–46.5	124	62	1.38	0.74–2.58	19	0.70	0.30–1.62	0.57	0.21–1.52
>46.5	119	57	1.39	0.62–3.12	25	1.39	0.49–3.99	1.11	0.33–3.73
p for trend				*0.32*			*0.92*		*0.75*
**Waist to hip ratio**									
≤0.82	93	25	Ref		18	Ref		Ref	
0.83–0.87	117	57	1.71	0.95–3.07	26	1.12	0.56–2.26	0.65	0.26–1.57
0.88–0.92	116	53	1.55	0.85–2.81	17	0.61	0.28–1.33	0.36	0.14–0.95
>0.92	144	70	1.61	0.90–2.90	39	1.26	0.63–2.50	0.63	0.27–1.50
p for trend				*0.27*			*0.63*		*0.38*
**Waist circumference (cm)**									
≤87.88	94	36	Ref		23	Ref		Ref	
87.89–97.75	118	39	0.88	0.48–1.60	25	0.93	0.45–1.92	1.14	0.48–2.73
97.76–110.25	121	56	1.30	0.68–2.48	25	1.11	0.48–2.57	0.95	0.36–2.45
>110.25	137	74	1.55	0.68–3.55	27	1.08	0.35–3.31	0.59	0.17–2.03
p for trend				*0.20*			*0.83*		*0.34*
**Hip circumference (cm)**									
≤103.18	99	47	Ref		24	Ref		Ref	
103.19–111.63	126	42	0.72	0.40–1.28	33	1.29	0.64–2.59	2.10	0.90–4.89
111.64–123.15	122	56	0.91	0.48–1.72	23	1.06	0.45–2.48	1.43	0.51–4.03
>123.15	123	60	0.73	0.31–1.73	20	1.00	0.31–3.29	1.89	0.45–7.98
p for trend				*0.65*			*0.81*		*0.61*

*BMI measured at interview.

*Abbreviations*: CO number of controls, CA number of cases, ER estrogen receptor, PR progesterone receptor.

Adjusted for age, ethnicity, country of origin (United States, Caribbean, other), education, family history of breast cancer, history of benign breast disease, age at menarche, age at menopause (for postmenopausal women), parity (continuous), breastfeeding (yes/no), age at first birth, HRT use (ever/never), OC use (ever/never). BMI also adjusted for waist circumference. Percent body fat, waist circumference, and hip circumference, also adjusted for BMI. For postmenopausal women, percent body fat adjusted for years since menopause instead of age at menopause.

## Discussion

In this study we found that BMI did not play a major role on breast cancer among AA women, except for a significant inverse association for ER-/PR- tumors in post-menopausal women. Furthermore, higher waist and hip circumferences increased pre-menopausal breast cancer risk, independent of BMI. While there was a suggestion of increased risk with higher fat mass and percent body fat among post-menopausal women, confidence intervals included the null value.

Very few studies have previously evaluated the role of recent BMI on breast cancer risk among AA (reviewed in [3,7,8]) and in general, previous findings are in agreement with ours. As in our study, no association with pre-menopausal breast cancer was reported in six other case–control studies [16-21] and one cohort study [22]. For post-menopausal breast cancer, results have been inconsistent. Similar to our results, no increased risk was reported in most of these studies, with an inverse association suggested in four of the case–control studies [16,18,23,24], weak and non-significant association in another case–control study [21] and in the Multi-Ethnic Cohort [25], and no association in the Black Women’s Health Study cohort [22]. Only two case–control studies have reported an increased risk among AA women [17,19]. Two additional studies conducted in Nigeria [26] and Barbados [27] reported no association with BMI. One of the most striking results from our study is the strong inverse association with BMI for ER-/PR- tumors among post-menopausal women. Similar inverse association was suggested in the two studies that presented stratified analyses by receptor status, the Women’s CARE Study [21] and in the Black Women’s Health Study [22]. However, confidence intervals in these two studies included the null, and, therefore, more studies are needed to understand the role of BMI on ER-/PR- tumors and on postmenopausal breast cancer in general.

In contrast to the findings for general obesity, we found a strong association with waist and hip circumferences, after adjusting for BMI in pre-menopausal women. While studies have typically used WHR to measure body fat distribution, waist circumference has been shown to be a better marker of central obesity than WHR in AA women [4]. Other studies, largely conducted among EA women, have generally reported increased risk of postmenopausal breast cancer associated with central adiposity, mostly measured with WHR [4], although there is some inconsistency across studies particularly for WHR [6] and no association for premenopausal breast cancer [4]. However, a recent meta-analysis reported that after adjusting for BMI, the association with WHR for postmenopausal breast cancer disappeared, while introducing an association for pre-menopausal breast cancer [9]. To our knowledge, only the Carolina Breast Cancer Study (CBCS) [18], the Black Women’s Health Study (BWHS) [22], the San Francisco Bay Area Breast Cancer Study (in pre-menopausal women) [20], and the Nigeria Breast Cancer Study (NBCS) [28] have reported on the association in AA women. For pre-menopausal breast cancer, two of the case–control studies (CBCS and NBCS) [18,28] found elevated risk with higher WHR [18,28] and waist circumference adjusted for BMI [28]. However, in contrast to our findings, an inverse association with hip circumference adjusted for BMI was found in the NBCS [28]. No association with WHR or waist circumference was found in the Bay Area case–control study [20] or the BWHS [22].

For post-menopausal breast cancer, the two case–control studies [18,28] suggested increased risk with central obesity. In CBCS [18], there was a suggestion of increased risk with higher WHR after adjusting for BMI but the confidence interval include the null, similar to our findings. The NBCS [28] found increased risk for high waist circumference and WHR independent of BMI, and an inverse association with hip circumference, which is in the same direction as our results. There was little evidence of an association with self-reported waist circumference or WHR in the BWHS [22].

To examine breast cancer relationships with obesity and central adiposity independently, our analytic strategy involved generating mutually adjusted risk estimates. Because measures of central obesity (e.g., waist circumference) and BMI in general tend to be correlated, however, there is potential for multicollinearity and inadvertent introduction of bias [29]. To ensure that this is not a concern, we carried out additional analyses to examine the impact of assessing BMI simultaneously with waist circumference, and determine whether issues with multicollinearity were impacting our risk estimates. Since the outcome is binary, the options for testing multicollinearity such as variance inflation factor (VIF) and tolerance, which are applied to continuous outcomes, do not directly apply. However, a general recommendation to check multicollinearity in logistic models is to run a similar model in linear regression and obtain collinearity statistics [30]. When we obtained tolerance and VIF statistics using this approach, the VIFs for BMI and waist circumference were over 4, indicating that the respective standard errors of these variables are twice as inflated relative to the absence of multicollinearity. However, when we checked the standard errors of these two variables in the logistic model, they were 0.01 for waist circumference and 0.02 for BMI, indicating that there was no substantial inflation, and suggesting that multicollinearity, if any, was not a severe issue in this model. Another sign of multicollinearity is if the global model is significant but none of the individual covariates are significant. In our model that included waist circumference and BMI, the coefficients of both variables were statistically significant (p ≤ 0.05). Lastly, we obtained significant associations both when waist circumference was treated in linear and non-linear forms. Put together, these results point to an issue of confounding rather than moderate or severe multicollinearity. In general, when waist circumference is included in the model as independent variables with BMI as a covariate, BMI becomes more of an index of lean mass than fatness in this multivariate model because the component of weight due to fatness is accounted for by abdominal circumference [31]. Therefore, the evaluation of waist circumference adjusted for BMI may be a better assessment of the association with adiposity. Given this, it makes sense that the effect of adjusting for BMI was more pronounced in premenopausal women, who tend to have more lean mass.

The role of body composition on breast cancer risk has received little attention, particularly among AA women and, to our knowledge, we are the first study to evaluate it and found no significant associations. It should be noted that we used bioelectrical impedance to measure body composition, which is not as precise as other methods, such as dual energy x-ray absorptiometry (DXA) and computed axial tomography (CAT) [32]. Therefore, our methods may have led to some degree of random misclassification in body composition measures that may have hampered our ability to detect an association. We are also aware that breast cancer treatment and menopause may affect body composition [32]. Increases in body fat and decreases in lean body mass after breast cancer treatment have been reported in studies, but not consistently, and these changes are not easily separated from those occurring with the aging process [32]. For example, it is not certain whether these changes in adiposity found in some studies could be attributed to chemotherapy, or could be due to ovarian failure caused by chemotherapy in premenopausal women [32] or to behavioral changes after breast cancer diagnosis. We adjusted analyses of body composition for age and years since menopause, but were not able to adjust for years since ovarian failure in pre-menopausal women.

Our findings generally confirm the small but growing body of literature suggesting that obesity may have a different impact on AA compared to EA women. The association of BMI with body fatness varies by age, gender, and race/ethnicity [33]. Body weight and BMI measures may not capture variations of fat mass and lean mass among women. In fact, for a given BMI, AA women tend to have higher lean mass and lower fat than white women [33]. Therefore, for AA women, waist and hip circumferences and percent body fat may reflect adiposity better than BMI and this may explain some of our findings. For example, higher waist circumference has been associated with insulin resistance and higher levels of IGF-I compared to general obesity, which is linked to higher levels of estrogens among postmenopausal women [9]. It is possible that central and general obesity play different roles in AA and EA because the relative role of hormonal pathways may be different in the two groups. For example, consistent with other studies, the Multi-Ethnic Cohort Study found lower postmenopausal breast cancer rates in AA compared to white women, but endogenous estrogen levels were found to be significantly higher among AA, suggesting that estrogens may play a less significant role on breast cancer etiology among them [34]. Furthermore, a cross-sectional study among AA pre-menopausal women found that upper body fat distribution (WHR > 0.8) was a better marker for a high risk hormonal profile (high estrogens, androgens, and prolactin, and low sex hormone binding globulin) for breast cancer than general obesity [35].

Case–control studies are subjected to well-known limitations such as recall bias and selection bias. It is well known that women tend to underestimate weight and overestimate height, which could affect BMI computation. However, for the main variables under investigation we did not rely on participants’ recall and, instead, objective body measurements were taken. Case–control studies are also prone to selection bias if the participating controls are not representative of the source population that gave rise to cases. To improve participation among AA controls, we supplemented RDD recruitment for AA women in NJ with community recruitment. Community-based recruitment was efficient in recruiting participants and even some cases and RDD controls who refused to participate in our study when they were first approached, called us to do it after they saw the study advertised in their communities (e.g., churches). This gave us some reassurance that the three groups (cases, RDD controls, and community controls) came from the same source population [11]. Another limitation in our study is that we did not have information on obesity-related comorbidities and, therefore, we were not able to adjust for them. To some extent, this may have explained the association found with abdominal obesity in premenopausal women.

## Conclusions

Our study contributes to the limited literature on BMI and other measures of adiposity and breast cancer risk in AA women. As more evidence accumulates on the relationship between BMI and breast cancer, it is becoming obvious that the association is not straightforward, with obesity affecting differently the various subgroups defined by age, race/ethnicity, hormone receptor status, and use of exogenous hormones. For AA women, the evidence remains scarce, but points to a different relationship from that reported in EA, suggesting possible different mechanisms. Larger studies in AA women are needed to further evaluate these issues, as well as evaluate the effect by “intrinsic” subtypes (luminal A, luminal B, basal-like and human epidermal growth factor receptor 2-positive/estrogen receptor-negative), which were suggested in one study to be affected differently by adiposity measures [36]. Future research is also needed to address the possibility that obesity affects breast cancer risk through its contribution to obesity-related comorbidities, including diabetes, which are more common among AA breast cancer patients, possibly through overlapping biologic mechanisms underlying associations between obesity, comorbidities, and breast cancer risk.

In summary, we found that higher waist and hip circumference were associated with increased risk of premenopausal breast cancer. While we did not find increased risk for general obesity, other studies have found that a higher BMI is associated with more advanced disease at diagnosis [37-39]. Therefore, maintenance of a healthy weight should be a goal for all women, but particularly for AA women. By doing so, they could improve their chances of survival if they develop breast cancer, as well as prevent other chronic diseases highly prevalent in this population, such as type II diabetes and cardiovascular disease [40].

## Abbreviations

AA: African ancestry; EA: European ancestry; NYC: New York City; NJ: New Jersey; RDD: Random digit dialing; SD: Standard deviation; OR: Odds ratio; CI: Confidence interval; BMI: Body mass index; WHR: Waist-to-hip ratio; HRT: Hormone replacement therapy; OC: Oral contraceptives; ER: estrogen receptor; PR: Progesterone receptor; NHANES: National Health and Nutrition Examination Survey; WCHS: Women’s Circle of Health Study; NJSCR: New Jersey State Cancer Registry.

## Competing interests

The authors declare that they have no competing interests.

## Authors’ contributions

EVB and CBA designed the study. EVB, CBA, GC directed its implementation. EVB and UC designed the analytic strategy. UC and GZ conducted data processing and cleaning. UC conducted statistical analyses. SEM and CCH provided expertise in the interpretation of results. EVB wrote the first draft of the manuscript and all co-authors provided substantive comments and editorial review and approved the final version of the manuscript.

## Pre-publication history

The pre-publication history for this paper can be accessed here:

http://www.biomedcentral.com/1471-2407/13/475/prepub
